# ATP6V1B1-Associated Inherited Distal Renal Tubular Acidosis in Children: Insights from a Literature Review

**DOI:** 10.3390/children13030436

**Published:** 2026-03-23

**Authors:** Andreea Liana Bot (Rachisan), Marius Cosmin Colceriu, Diana Jecan-Toader, Bogdan Bulata, Dan Delean, Mihaela Sparchez

**Affiliations:** 1 Faculty of Nursing and Health Sciences, Department 2, University of Medicine and Pharmacy “Iuliu Hatieganu”, 400023 Cluj-Napoca, Romania; 2 Department of Pediatric Nephrology, Cluj-Napoca Children’s Hospital Gheorghieni, 400023 Cluj-Napoca, Romania; 3 Department of Pediatrics II, University of Medicine and Pharmacy “Iuliu Hatieganu”, 400023 Cluj-Napoca, Romania; 4 No 8 Mother and Child, Department of Pediatrics II, University of Medicine and Pharmacy “Iuliu Hatieganu”, 400023 Cluj-Napoca, Romania

**Keywords:** distal renal tubular acidosis, ATP6V1B1, children, inherited kidney disease, nephrocalcinosis, hearing loss, genotype–phenotype correlation

## Abstract

Inherited distal renal tubular acidosis (dRTA) is a rare but clinically significant disorder of renal acid–base regulation that frequently presents in infancy or early childhood. Among the genetic causes of autosomal recessive dRTA, mutations in the ATP6V1B1 gene are particularly important due to their association with early-onset disease and sensorineural hearing loss. Failure to recognize and treat this condition promptly can result in growth retardation, bone disease, nephrocalcinosis, chronic kidney disease, and permanent auditory impairment. This article presents a comprehensive review of the pediatric literature concerning dRTA. We focus on the pathophysiology, pediatric presentation, renal and audiological outcomes, genetic architecture, and management implications of ATP6V1B1-associated dRTA in children. We highlight evolving genotype–phenotype correlations, the emerging recognition of autosomal recessive disease mechanisms, and the importance of early diagnosis and long-term multidisciplinary follow-up.

## 1. Introduction

Distal renal tubular acidosis (dRTA) is a disorder of impaired urinary acidification resulting from dysfunction of acid-secreting mechanisms in the distal nephron. This defect leads to chronic hyperchloremic metabolic acidosis with a normal anion gap and an inability to appropriately lower urine pH. In children, sustained metabolic acidosis has wide-ranging systemic consequences, including impaired linear growth, disturbances in calcium and phosphate homeostasis, nephrocalcinosis, and progressive renal and skeletal complications [1,2]. Inherited forms of dRTA are genetically heterogeneous and most often present during infancy or early childhood. Pathogenic variants have been identified in several genes encoding proteins critical for distal tubular acid secretion, including ATP6V1B1, ATP6V0A4, SLC4A1, FOXI1, and WDR72 [3,4,5]. Among these, ATP6V1B1 is of relevance in pediatric nephrology because of its strong association with early-onset disease and extra-renal manifestations [3,6]. The ATP6V1B1 gene encodes the B1 subunit of the vacuolar H^+^-ATPase (V-ATPase), a multisubunit proton pump expressed on the apical membrane of α-intercalated cells in the collecting duct. This enzyme complex is essential for active hydrogen ion secretion, enabling renal acid excretion and maintenance of systemic acid–base homeostasis. Loss of B1 subunit function disrupts proton transport, leading to failure of urinary acidification and the characteristic biochemical phenotype of dRTA [3,4]. Beyond the kidney, ATP6V1B1 is also expressed in epithelial cells of the inner ear, where it contributes to endolymphatic acid–base regulation. This dual expression provides a biological explanation for the long-recognized association between ATP6V1B1-related dRTA and sensorineural hearing loss (SNHL). Early molecular studies firmly established ATP6V1B1 as a cause of autosomal recessive dRTA associated with congenital or early-onset deafness, shaping diagnostic paradigms for more than two decades [3,6]. Subsequent clinical series and longitudinal observations revealed substantial phenotypic variability. Hearing loss may be absent in early childhood or develop progressively over time, and renal disease severity varies widely, even among individuals carrying identical pathogenic variants [5,7]. These findings suggested that modifier genes, environmental influences, and treatment timing may significantly affect disease expression and long-term outcomes. Further refinement of genotype–phenotype correlations emerged from multicenter pediatric cohorts, which demonstrated that nephrocalcinosis, growth impairment, and renal functional decline are not exclusive to a single genetic subtype and may overlap across different forms of inherited dRTA [4,8]. These observations highlighted the limitations of phenotype-based diagnostic approaches and reinforced the importance of molecular confirmation. More recently, advances in molecular genetics have expanded the clinical spectrum of ATP6V1B1-associated disease. The identification of atypical inheritance patterns and milder phenotypes has challenged the traditional view that ATP6V1B1 mutations invariably cause severe recessive disease with early deafness. These discoveries underscore the evolving complexity of dRTA genetics and the need for careful interpretation of genetic results in clinical practice [9]. Given that ATP6V1B1-associated dRTA is predominantly a pediatric-onset condition requiring lifelong therapy and surveillance, a child-centered synthesis of available evidence is essential. This review emphasizes the clinical importance of ATP6V1B1-related dRTA through a comprehensive review of pediatric studies, integrating classical pathophysiology with emerging genetic and phenotypic insights.

## 2. Molecular and Pathophysiological Basis

The vacuolar H^+^-ATPase (V-ATPase) is a highly conserved, multi-subunit enzyme complex responsible for proton transport across intracellular and plasma membranes. In the kidney, it is expressed on the apical membrane of α-intercalated cells in the collecting duct, where it mediates final urinary acidification [10,11]. The enzyme consists of a cytoplasmic V1 domain, which is responsible for ATP hydrolysis, and a membrane-bound V0 domain, which mediates proton translocation across the membrane [12]. ATP6V1B1 encodes the B1 subunit of the V1 domain. This subunit is tissue-specific, with predominant expression in the kidney and inner ear [3,6]. Loss or dysfunction of the B1 subunit impairs proper assembly and stability of the V-ATPase complex, leading to reduced proton secretion into the tubular lumen. Therefore, affected individuals are unable to appropriately acidify urine despite the presence of systemic metabolic acidosis [4]. To preserve electroneutrality in the extracellular fluid, the reduction in negatively charged bicarbonate ions is compensated by an increase in chloride (Cl^−^) reabsorption in the renal tubules. This relative retention of chloride maintains the balance between cations and anions in plasma. As a result, serum chloride levels rise, producing hyperchloremia. Hypokalemia is a common biochemical feature of dRTA and results from alterations in distal tubular ion transport secondary to impaired hydrogen ion secretion. In the collecting duct, defective function of the α-intercalated cells reduces the ability to secrete H^+^ via the apical H^+^-ATPase and H^+^/K^+^-ATPase transporters. This defect disrupts normal acid excretion and alters the electrochemical gradients within the distal nephron. To maintain sodium reabsorption, increased sodium delivery to the distal nephron enhances electrogenic sodium uptake through epithelial sodium channels (ENaCs) in principal cells. This process generates a more negative luminal potential, which favors potassium secretion through apical potassium channels (ROMK and BK channels). As a consequence, urinary potassium excretion increases. Additionally, chronic metabolic acidosis and secondary activation of the renin–angiotensin–aldosterone system may further stimulate distal sodium reabsorption and potassium secretion. The combined effect of enhanced distal potassium secretion and impaired H^+^/K^+^ exchange contributes to renal potassium wasting and the development of hypokalemia in dRTA.

**Figure 1:**
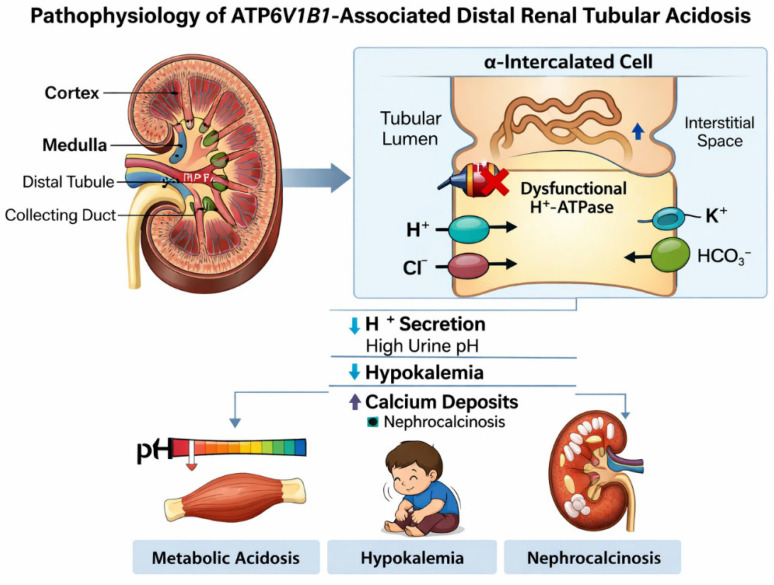
Pathophysiology of ATP6V1B1-Associated Distal Renal Tubular Acidosis. Schematic illustration showing impaired hydrogen ion secretion in α-intercalated cells of the distal nephron due to dysfunctional vacuolar H^+^-ATPase caused by ATP6V1B1 mutation, resulting in metabolic acidosis, hypokalemia, and nephrocalcinosis.

In the inner ear, V-ATPase activity contributes to regulation of endolymph composition and pH, which are essential for normal cochlear and vestibular function [13,14]. Disruption of proton transport in this setting is thought to impair inner ear homeostasis, resulting in sensorineural hearing loss and structural abnormalities such as enlarged vestibular aqueducts. The dual expression of ATP6V1B1 in renal and auditory epithelia thus provides a mechanistic explanation for the combined renal and auditory manifestations observed in affected children [3,15] (see Figure 1).

## 3. Clinical Presentation in Children

ATP6V1B1-associated distal renal tubular acidosis (dRTA) most commonly presents during infancy or early childhood, although delayed diagnosis into later childhood or adolescence has been reported. Early clinical manifestations are often non-specific and include poor feeding, recurrent vomiting, dehydration episodes, polyuria, polydipsia, and failure to thrive. These symptoms reflect the systemic effects of chronic metabolic acidosis as well as associated renal concentrating defects [8,16]. Growth impairment is a prominent early feature and may represent the primary concern prompting medical evaluation. In some children, dRTA is identified incidentally during assessment of nephrocalcinosis on imaging performed for unrelated indications or through family screening following diagnosis in an affected sibling [4,17]. Biochemically affected children demonstrate a characteristic pattern of normal anion gap metabolic acidosis with hyperchloremia. Serum bicarbonate levels are persistently reduced, urine pH remains inappropriately elevated, and hypokalemia is frequently observed, although severity varies. These laboratory features remain central to diagnosis and guide subsequent genetic evaluation [16,18] (Table 1).

## 4. Renal Manifestations and Outcomes

Renal complications are a major source of morbidity in ATP6V1B1-associated dRTA. Nephrocalcinosis is among the most consistent findings and is frequently present at the time of diagnosis. Chronic metabolic acidosis promotes bone buffering with increased calcium mobilization, while hypocitraturia reduces urinary inhibition of crystal formation, together predisposing to calcium phosphate deposition within the renal parenchyma [15,16]. Nephrolithiasis may develop later in childhood or adolescence and can contribute to pain, hematuria, and recurrent urinary tract infections. The severity of nephrocalcinosis varies widely and is influenced by age at diagnosis, adequacy of metabolic control, and long-term adherence to alkali therapy [4,18]. Growth failure and skeletal disease remain key concerns in pediatric patients. Persistent acidosis disrupts bone mineralization and interferes with growth hormone and insulin-like growth factor signaling. Untreated or inadequately treated children may develop rickets or osteomalacia, delayed motor development, and reduced final height. Early initiation and sustained optimization of alkali therapy are consistently associated with improved growth outcomes [15,19]. Most children maintain preserved glomerular filtration rate during early life; however, progression to chronic kidney disease has been described, particularly in those with severe nephrocalcinosis, recurrent stone disease, or delayed diagnosis. Long-term renal prognosis is therefore closely linked to early recognition and sustained metabolic control [4,16].

## 5. Hearing Phenotype and Audiological Findings

Sensorineural hearing loss (SNHL) is a defining feature of classical ATP6V1B1-associated dRTA but demonstrates considerable variability in onset and severity. Hearing impairment may be congenital, develop in early childhood, or present progressively during later childhood or adolescence. Importantly, hearing loss may be absent at the time of renal diagnosis [17,18]. Recent cohort analyses and expert reviews indicate that hearing loss is typically bilateral and progressive, although fluctuating thresholds have been reported. These findings underscore the importance of repeated audiological assessments rather than reliance on a single baseline evaluation [16,18]. Inner ear imaging frequently reveals enlarged vestibular aqueducts in affected individuals. This structural abnormality is associated with progressive hearing loss and increased susceptibility to sudden deterioration following minor head trauma or barotrauma. Recognition of this association has important implications for counseling families regarding activity precautions and the need for prompt evaluation of hearing changes [15,18].

## 6. Genetics and Inheritance Patterns

Traditionally, ATP6V1B1-associated dRTA has been classified as an autosomal recessive disorder. Most pediatric cases reported involve biallelic pathogenic variants, including truncating and deleterious missense mutations, and are typically associated with early-onset disease and a high prevalence of hearing impairment [4,17]. Large contemporary cohorts have demonstrated substantial phenotypic overlap between ATP6V1B1 and other dRTA-associated genes, highlighting the limitations of clinical features alone in predicting genotype. As a result, comprehensive genetic testing has become a cornerstone of diagnostic evaluation and family counseling in children with dRTA [17,18]. More recently, an autosomal dominant form of ATP6V1B1-associated dRTA has been described, caused by heterozygous variants affecting the Arg394 residue. Functional studies support a dominant-negative mechanism and affected individuals typically present with renal manifestations of dRTA but often lack significant hearing involvement. This emerging inheritance pattern challenges long-standing diagnostic assumptions and has important implications for genetic counseling [18,20].

## 7. Management and Follow-Up in Children

**Figure 2:**
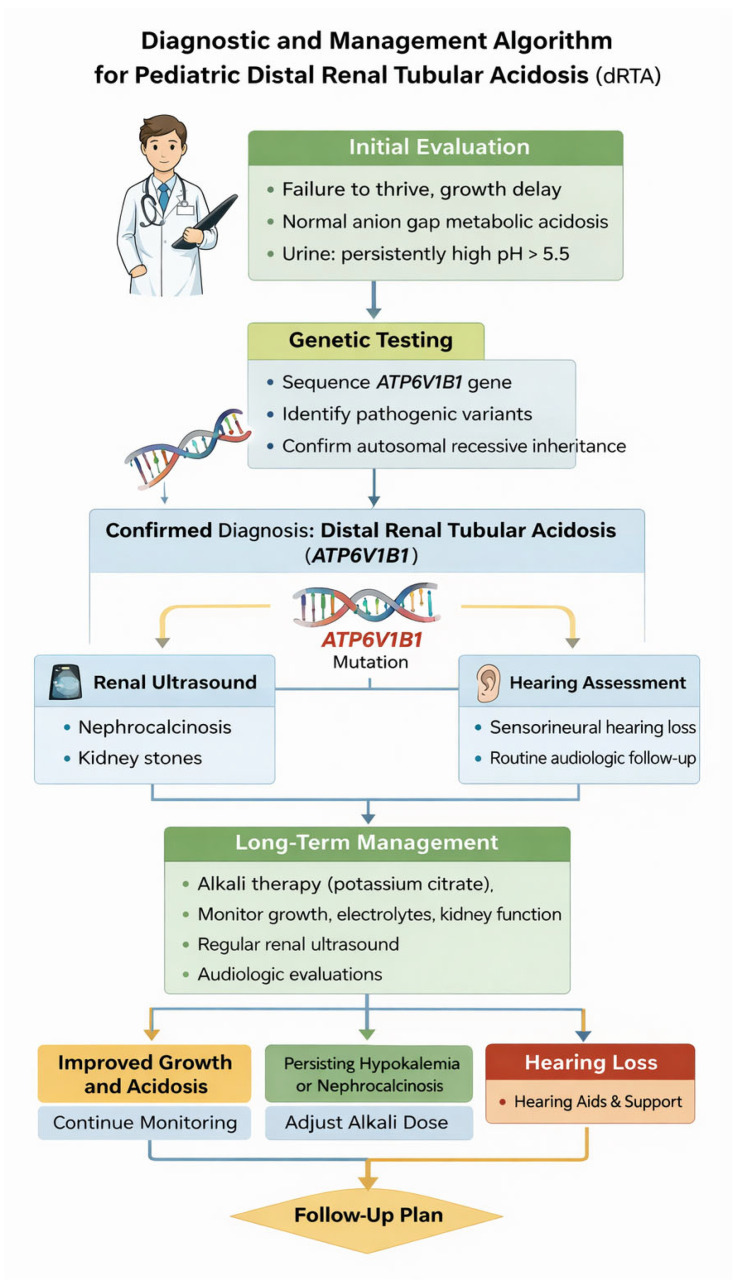
Diagnostic and Management Algorithm for Pediatric Distal Renal Tubular Acidosis. Flowchart outlining the approach to diagnosis of dRTA in children, including laboratory evaluation, genetic testing, renal imaging, hearing assessment, and long-term management.

Lifelong alkali therapy remains the cornerstone of management for ATP6V1B1-associated dRTA. The primary therapeutic agents are potassium citrate and sodium bicarbonate, which correct systemic metabolic acidosis, prevent hypokalemia, and increase urinary citrate excretion. Potassium citrate is often preferred in pediatric patients, as it addresses both potassium deficiency and urinary citrate deficiency, thereby reducing the risk of nephrocalcinosis and nephrolithiasis. The primary objectives of treatment are to: (1) normalize serum bicarbonate and maintain systemic acid–base homeostasis, (2) correct and prevent electrolyte abnormalities, particularly hypokalemia and hyperchloremia, (3) reduce renal complications, including nephrocalcinosis and nephrolithiasis, and (4) promote normal growth and bone health, preventing rickets or osteomalacia associated with chronic acidosis. Alkali therapy should be individualized and frequently adjusted during periods of rapid growth, intercurrent illness, or dehydration. Recommended doses typically range from 2–5 mEq/kg/day, divided into multiple daily doses, for both potassium citrate and sodium bicarbonate. Dose titration should be guided by serum bicarbonate, potassium, urine pH, and clinical response [16,19] (Table 2). Children require regular follow-up with comprehensive assessment of growth parameters (height, weight, BMI) to adjust therapy and detect failure to thrive, laboratory tests, including serum electrolytes, bicarbonate, and acid–base status. Renal imaging to monitor is necessary for nephrocalcinosis or nephrolithiasis and urinary parameters, such as urine pH and citrate excretion, to guide therapy efficacy [16]. Due to the high prevalence and progressive nature of sensorineural hearing loss in ATP6V1B1-associated dRTA, baseline and serial audiological evaluations are mandatory. Early involvement of audiology and otolaryngology services facilitates timely interventions, including hearing aids or cochlear implantation. Structural evaluation with inner ear imaging, such as MRI or CT; may identify abnormalities like an enlarged vestibular aqueduct, which is associated with progressive hearing impairment [18]. Optimal management is inherently multidisciplinary, involving pediatric nephrologists in order to coordinate metabolic control, growth monitoring, and renal imaging, audiologists and otolaryngologists: to manage hearing assessment and interventions. The dietitians can provide guidance on caloric intake, micronutrients, and electrolyte balance, and the genetic counselors can advise families regarding inheritance patterns, recurrence risks, and available genetic testing [18,20] (see Figure 2).

## 8. Emerging Insights and Genotype–Phenotype Complexity

Distal renal tubular acidosis (dRTA) is a rare but clinically significant disorder in children, characterized by impaired urinary acidification leading to metabolic acidosis, nephrocalcinosis, growth failure, and long-term risk of chronic kidney disease (CKD). Over the past two decades, advances in molecular genetics and collaborative cohort studies have substantially refined understanding of the disease spectrum, particularly with respect to ATP6V1B1-associated dRTA. The studies summarized in Table 3 collectively illustrate an evolving paradigm in which genotype–phenotype correlations are increasingly nuanced, inheritance patterns more complex, and management strategies more individualized [16,18]. Early descriptions of ATP6V1B1-related dRTA emphasized a classic autosomal recessive phenotype combining severe early-onset metabolic acidosis with sensorineural hearing loss (SNHL). However, large cohort and case series data have challenged this rigid framework. Palazzo et al. demonstrated that renal disease severity overlaps considerably across genotypes, including ATP6V1B1, ATP6V0A4, and SLC4A1, thereby limiting the predictive value of clinical features alone [17]. Importantly, hearing loss was not universally present at diagnosis, underscoring the need for longitudinal surveillance rather than reliance on baseline audiologic assessment [17]. The significance of intrafamilial and interindividual variability is further highlighted by the multicenter pediatric series reported by Subaşıoğlu Uzak et al., which expanded the mutational spectrum of ATP6V1B1 and documented heterogeneous renal and auditory manifestations even among siblings [21].

These observations reinforce the concept that modifying genetic, epigenetic, or environmental factors likely influence disease expression. Such variability complicates early prognostication but supports the growing consensus that early genetic testing is essential for accurate diagnosis, counseling, and anticipatory guidance [21]. Recent population-specific genotype–phenotype studies have contributed valuable regional insights. Hammi et al. characterized the genetic architecture of pediatric dRTA in Tunisia, identifying a predominance of ATP6V1B1 and ATP6V0A4 variants and emphasizing early-onset nephrocalcinosis and hypokalemia as common features [22]. Together, this study underscores the importance of considering ethnic and regional mutation patterns while maintaining broadly applicable clinical surveillance strategies. A particularly important development in the field is the recognition of autosomal dominant ATP6V1B1-associated dRTA. Daenen et al. described heterozygous Arg394 variants causing dRTA with a milder renal phenotype and minimal auditory involvement, fundamentally challenging the long-standing assumption that ATP6V1B1 disease is exclusively recessive and strongly linked to SNHL [20]. This discovery has immediate implications for genetic counseling, family screening, and differential diagnosis in children presenting with incomplete or atypical dRTA phenotypes. The inclusion of this inheritance pattern in the updated GeneReviews synthesis reflects the rapid integration of new molecular insights into clinical practice [18]. The auditory phenotype associated with ATP6V1B1 variants remains a defining but variably expressed feature. The audiologic and imaging series by Joshua et al. provided early evidence linking ATP6V1B1-associated dRTA with progressive SNHL and a high prevalence of enlarged vestibular aqueduct (EVA), suggesting a shared pathophysiologic mechanism affecting proton transport in both renal and inner ear epithelia [15]. Subsequent studies have refined this association, indicating that while SNHL is common, its onset may be delayed and its severity variable [15]. Consequently, contemporary guidelines emphasize routine and repeated hearing assessments rather than single-time-point evaluations [18]. From a management perspective, the ERKNet/ESPN clinical practice points articulated by Trepiccione et al. represent a milestone in standardizing pediatric dRTA care [16]. These recommendations stress early initiation of alkali therapy, aggressive correction of metabolic acidosis to prevent nephrocalcinosis and growth impairment, and systematic monitoring for CKD and hearing loss. Importantly, these guidelines acknowledge genotype-informed risk stratification while cautioning against overreliance on genotype alone to predict outcomes [18]. Therapeutic advances and optimization of long-term management are comprehensively reviewed by Boyer et al., who highlights recent developments in alkali formulations, individualized dosing strategies, and adherence-focused interventions [19]. Their review reinforces evidence that early and sustained metabolic control can significantly improve growth outcomes and renal prognosis, even in genetically severe forms of dRTA. These findings align with cohort data indicating that delayed diagnosis and suboptimal treatment are major determinants of adverse outcomes, sometimes outweighing the impact of genotype [19]. Collectively, these studies point toward an integrated model of pediatric dRTA care in which genetic diagnosis, clinical monitoring, and individualized therapy are tightly interwoven. While molecular testing has become indispensable for diagnosis and counseling, it does not obviate the need for vigilant longitudinal follow-up. Renal and auditory phenotypes may evolve over time, and emerging data on dominant inheritance and variable expressivity further complicate simplistic genotype–phenotype assumptions [17,20].

Despite substantial progress in understanding ATP6V1B1-associated dRTA, current evidence has several limitations. Most studies are observational, with relatively small sample sizes and geographically restricted cohorts, which limits generalizability and the ability to draw firm genotype–phenotype conclusions. Heterogeneity in diagnostic criteria, follow-up duration, and outcome reporting further complicates cross-study comparisons [17,21,22]. Predicting clinical outcomes based solely on genotype remains challenging. While certain ATP6V1B1 variants are recurrent, disease severity, and risk of CKD vary widely, even among siblings sharing the same mutation [21]. This intrafamilial variability suggests that additional genetic, epigenetic, or environmental factors significantly modulate disease expression, underscoring the need for individualized monitoring and therapy [18,19].

Future research priorities should focus on expanding multicenter, ethnically diverse cohorts with standardized longitudinal data collection to improve predictive models. Identification of reliable biomarkers for early renal and auditory involvement could enhance risk stratification and guide timely intervention. Additionally, emerging precision medicine strategies, including gene therapy and targeted molecular interventions, hold promise for altering disease progression, particularly in severe or early-onset cases [19]. Integration of multi-omics approaches may also elucidate modifiers of expressivity and inform personalized therapeutic strategies. Combining genotype-informed management with routine renal and audiologic surveillance currently represents best practice and provides a foundation for future precision medicine approaches in pediatric dRTA.

## 9. Conclusions

Contemporary literature emphasizes a shift from a gene-centered to a patient-centered view of ATP6V1B1-associated dRTA. Early recognition and prompt alkali therapy are essential to prevent growth failure, nephrocalcinosis, and CKD. Genetic testing facilitates accurate diagnosis, family counseling, and identification of atypical inheritance patterns, including dominant variants. Audiologic manifestations may progress over time, requiring regular monitoring. Key research gaps still remain: genotype–phenotype correlations are imperfect, disease modifiers are poorly understood, and long-term treatment strategies lack robust evidence. Future studies should focus on large, prospective registries, predictive biomarkers, and multi-omics approaches. Precision medicine, including gene-targeted therapies, may offer new treatment avenues. Addressing these gaps will optimize individualized care and improve long-term outcomes for affected children.

## Figures and Tables

**Table 1:** Clinical and Biochemical Features of ATP6V1B1-Associated. Distal Renal Tubular Acidosis in Children.

Category	Domain	Typical Findings in Children with Distal Renal Tubular Acidosis
**Clinical**	Age at presentation/onset	Infancy to early childhood (occasionally later in childhood)
	Growth	Failure to thrive and short stature
	Hearing	Sensorineural hearing loss, often congenital or progressive
**Biochemical**	Acid–base status	Hyperchloremic metabolic acidosis with normal anion gap
	Serum electrolytes	Hypokalemia and hyperchloremia
	Urine pH	Inappropriately high (>5.5) despite systemic metabolic acidosis
**Renal manifestations**	Nephrocalcinosis	Very common finding on renal imaging
	Nephrolithiasis	May develop with increasing age
**Skeletal**	Bone involvement	Rickets in children; osteomalacia in severe or prolonged untreated cases
**Imaging (ear)**	Inner ear imaging	Enlarged vestibular aqueduct may be present
**Genetic**	Inheritance	Most commonly autosomal recessive; autosomal dominant forms reported (e.g., Arg394 variants)
**Treatment response**	Response to therapy	Improvement in growth and metabolic control with alkali therapy

**Table 2:** Management of ATP6V1B1-Associated Distal Renal Tubular Acidosis.

Category	Intervention/Medication	Typical Dose/Notes	Monitoring/Follow-Up
**Alkali therapy**	Potassium citrate	2–5 mEq/kg/day divided 3–4 times daily	Serum potassium, bicarbonate, urine pH, growth parameters
**Alkali therapy**	Sodium bicarbonate	2–5 mEq/kg/day divided 3–4 times daily; usually reserved if potassium citrate not tolerated	Serum electrolytes, acid–base status, kidney function
**Electrolyte management**	Potassium supplements (if persistent hypokalemia)	Individualized, oral potassium salts as needed	Serum potassium, ECG if severe
**Renal monitoring**	Renal imaging	Ultrasound every 6–12 months initially	Assess nephrocalcinosis or nephrolithiasis progression
**Growth monitoring**	Anthropometry	Height, weight, and BMI at every clinic visit	Adjust alkali dosing during periods of rapid growth
**Audiology**	Hearing assessment	Baseline audiogram at diagnosis; repeat every 6–12 months	Early identification of sensorineural hearing loss; facilitate timely hearing aids/cochlear implantation
**Multidisciplinary care**	Pediatric nephrology	Ongoing coordination of metabolic control	Periodic review of labs, growth, kidney function
	Audiology/Otolaryngology	Baseline and serial evaluations	Intervention planning for hearing loss
	Dietitian	Guidance on adequate nutrition	Ensure sufficient caloric and micronutrient intake
	Genetic counseling	At diagnosis and family planning	Explain inheritance patterns, risks, and available testing

**Table 3:** Key clinical evidence informing ATP6V1B1-associated inherited distal renal tubular acidosis in children.

First Author (Year)	Study Design	Pediatric Population	Genetic Findings	Renal Phenotype	Hearing Phenotype	Key Contributions to the Field
Alexander et al. (GeneReviews, updated 2025) [18]	Expert synthesis	Yes	Recessive and dominant *ATP6V1B1* variants	Classic distal RTA spectrum	SNHL common but variable	Comprehensive synthesis of pediatric presentation, genetics, and management; incorporated emerging autosomal dominant inheritance
Daenen et al. (2025) [20]	Mechanistic and clinical genetics study	Mixed (families including children)	Heterozygous Arg394 variants	dRTA without severe early nephrocalcinosis	Hearing loss uncommon	Identified novel autosomal dominant *ATP6V1B1*-dRTA mechanism with reduced auditory involvement
Boyer et al. (2024) [19]	Narrative therapeutic review	Yes	Multiple genetic causes of pediatric dRTA	Growth failure, nephrocalcinosis, CKD risk	Hearing loss acknowledged in genetic forms	Reviewed recent advances in alkali therapy, individualized dosing, and long-term pediatric outcomes; emphasized early diagnosis and adherence
Hammi et al. (2023) [22]	National genotype–phenotype cohort	Yes	Predominantly *ATP6V1B1* and *ATP6V0A4* variants	Early-onset nephrocalcinosis, hypokalemia	Variable SNHL	Characterized dRTA genetics in a Tunisian pediatric population; reinforced ethnic and regional mutation patterns
Trepiccione et al. (2021) [16]	ERKNet/ESPN clinical practice points	Yes	Multiple dRTA genes including *ATP6V1B1*	Nephrocalcinosis; CKD risk	Hearing surveillance recommended	Established pediatric-focused diagnostic, treatment, and monitoring recommendations
Palazzo et al. (2017) [17]	Large international cohort	Yes (mixed ages; pediatric majority)	*ATP6V1B1* among multiple dRTA genes	Overlapping renal severity across genotypes	Hearing loss not universal at presentation	Demonstrated limitations of phenotype-based genetic prediction; supported early genetic testing
Subaşıoğlu Uzak et al. (2013) [21]	Multicenter case series	Yes	Homozygous and compound heterozygous variants	Nephrocalcinosis, hypokalemia, growth retardation	Variable SNHL	Expanded pediatric mutation spectrum; highlighted intrafamilial phenotypic variability
Joshua et al. (2008 [15])	Audiology and imaging series	Yes	*ATP6V1B1*-associated dRTA	Not primary focus	Progressive SNHL; frequent EVA	Linked *ATP6V1B1*-dRTA with enlarged vestibular aqueduct and progressive hearing loss

## Data Availability

The original contributions presented in this study are included in the article. Further inquiries can be directed to the corresponding authors.
